# A Need to Study the Immune Status of Frail Older Adults

**DOI:** 10.1186/1742-4933-3-1

**Published:** 2006-01-19

**Authors:** Steven C Castle, Koichi Uyemura, Tamas Fulop, Katsuiku Hirokawa, Takashi Makinodan

**Affiliations:** 1 Geriatric Research, Education and Clinical Center (GRECC), VA Greater Los Angeles Healthcare System and Multicampus Division of Geriatric and Gerontology, Department of Medicine, UCLA, Los Angeles, CA 90073; 2 Research Center on Aging, Geriatric Division, Faculty of Medicine, University of Sherbrooke, Sherbrooke, Quebec, Canada; 3 Institute of Applied Medicine, Tokyo, Japan

The frail older adult subpopulation, which is growing at a rapid rate, contributes significantly to the increasing global healthcare cost [1]. Many frail older adults are immunologically compromised, and, therefore, succumb to common infections in spite of the availability of antibiotics and other therapeutic measures. It is also not known how repeated or recurrent infections affect the progression of the underlying chronic disease. Thus, there is an urgent need to understand the nature of the diminished immune capacity of frail older adults, for it could provide new insight into developing effective intervention modalities. Unfortunately, very little research has been done to describe and elucidate the immune deficits of the frail older adults. In the past, researchers have been discouraged to study this subpopulation because of its complexity in terms of disease category and disease burden.

In contrast, much is known of the immune status of the healthy older adult subpopulation, for this subpopulation has been investigated extensively over the past three decades. Thus, it is known that T cell-dependent immune functions decline with age [2], and associated with the decline are structural changes in T cells [3]. However, a review of more than 200 scientific articles that evaluated healthy older adults, who were selected on a set of rigorous criteria as defined by the SENEIUR Protocol [4], showed that the magnitude of decline in T cell-dependent immune functions with age is modest [5], relative to that of the aging mouse model [6]. More recently, Sehl and Yates [7] analyzed changes in various physiologic functions with age from 469 studies involving more than 54,000 healthy and frail older adults. The expansive review included 43 immunologic studies of 372 individuals. They found that the mean annual rate of decline with age in immune functions is greater than that of other physiologic functions that were assessed. The authors concluded that the deterioration in immune function in older adults is due not only to aging, but also the presence of chronic disease. This review also underscores the need to evaluate the immune status of frail older adults with chronic diseases.

Recently, the influence of chronic disease on T cell immunity as been investigated [8], using the Cumulative Illness Rating Scale (CIRS) [9]. CIRS is an instrument that measures disease burden in individuals with various chronic diseases, but with no evidence of acute deterioration or infection. The CIRS instrument was originally developed in 1968 and is acknowledged as a user-friendly, comprehensive review of medical problems of 14 organ systems [9]. It is based on a 0 to 4 rating of each organ system. The scale has been validated in older adults living in long-term care facilities and congregate apartments in the community and has demonstrated better validity in predicting healthcare outcomes than functional measures [10]. T cell immunity was based on phytohemagglutinin (PHA)-induced proliferation and production of immunosuppressive interleukin (IL)-10 and immunoenhancing IL-12. The study showed that decrease in T cell proliferation, increase in production of IL-10 and decrease in production of IL-12 are linearly correlated with increase in chronic disease burden (i.e., increased CIRS score), but not with increase in chronologic age, between 51 to 95 years.

The demonstration that reduced immunity in older adults is correlated with chronic disease burden, but not with chronologic age, suggests that chronic disease burden markedly enhances the reduction in immunity of older adults caused by biologic aging. Others have suggested chronic infections, caused by especially cytomegalovirus, and also by *Helicobacter pylori, Mycobacterium tuberculosis, Chlamydia pneumoniae, Herpes *viruses, *Epstein-Barr *virus, and *Hepatitis *viruses, could play a role on progression of chronic disease, especially atherosclerosis, and impaired immunity [11-15]. While CIRS does not directly address prior viral infections, if these infections do impact on progression of disease it would be predicted that there would be a correlation between CIRS and evidence of chronic infection. Chronic infections, increased levels of inflammatory mediators, disease progression and frailty have a very complex association, and, furthermore, an unclear temporal relationship. Therefore, at this stage of progress, it would be appropriate and timely, to study the immune status of frail older adults using an instrument, such as the CIRS, to categorize frail older adults according to specific chronic disease and disease burden. The immune status of frail older adults in each category could then be assessed, and immunosuppressive factors produced by specific disease that are present in the microenvironment of immune cells could be identified and their impact on immunity mitigated. Consequently, the compromised immune status of frail older adults could be boosted to that of healthy older adults, thereby improving their innate and adaptive immunologic defense mechanisms to infections and response to vaccination. This should significantly increase their resistance to infectious and other immunocompromised-related diseases, possibly slow the progression of chronic diseases, and, therefore, contribute to the goal of reducing the global healthcare cost.
